# Malaria Risk Stratification and Modeling the Effect of Rainfall on Malaria Incidence in Eritrea

**DOI:** 10.1155/2019/7314129

**Published:** 2019-04-01

**Authors:** Meron Mehari Kifle, Tsega Tekeste Teklemariam, Adam Mengesteab Teweldeberhan, Eyasu Habte Tesfamariam, Amanuel Kidane Andegiorgish, Eyob Azaria Kidane

**Affiliations:** ^1^Asmara College of Health Sciences, School of Public Health, Department of Epidemiology and Biostatistics, Asmara, Eritrea; ^2^Ministry of Health, Asmara, Eritrea

## Abstract

**Background:**

Malaria risk stratification is essential to differentiate areas with distinct malaria intensity and seasonality patterns. The development of a simple prediction model to forecast malaria incidence by rainfall offers an opportunity for early detection of malaria epidemics.

**Objectives:**

To construct a national malaria stratification map, develop prediction models and forecast monthly malaria incidences based on rainfall data.

**Methods:**

Using monthly malaria incidence data from 2012 to 2016, the district level malaria stratification was constructed by nonhierarchical clustering. Cluster validity was examined by the maximum absolute coordinate change and analysis of variance (ANOVA) with a conservative post hoc test (Bonferroni) as the multiple comparison test. Autocorrelation and cross-correlation analyses were performed to detect the autocorrelation of malaria incidence and the lagged effect of rainfall on malaria incidence. The effect of rainfall on malaria incidence was assessed using seasonal autoregressive integrated moving average (SARIMA) models. Ljung–Box statistics for model diagnosis and stationary *R*-squared and Normalized Bayesian Information Criteria for model fit were used. Model validity was assessed by analyzing the observed and predicted incidences using the spearman correlation coefficient and paired samples *t*-test.

**Results:**

A four cluster map (high risk, moderate risk, low risk, and very low risk) was the most valid stratification system for the reported malaria incidence in Eritrea. Monthly incidences were influenced by incidence rates in the previous months. Monthly incidence of malaria in the constructed clusters was associated with 1, 2, 3, and 4 lagged months of rainfall. The constructed models had acceptable accuracy as 73.1%, 46.3%, 53.4%, and 50.7% of the variance in malaria transmission were explained by rainfall in the high-risk, moderate-risk, low-risk, and very low-risk clusters, respectively.

**Conclusion:**

Change in rainfall patterns affect malaria incidence in Eritrea. Using routine malaria case reports and rainfall data, malaria incidences can be forecasted with acceptable accuracy. Further research should consider a village or health facility level modeling of malaria incidence by including other climatic factors like temperature and relative humidity.

## 1. Background

Despite the drastic decline in morbidity and mortality over the past two decades, malaria continues to remain a global public health concern. In 2015, there were an estimated 212 million malaria cases globally, translating into an incidence rate of 94 per 1000 persons at risk—a 41% decrease from the rate in 2000 [1]. Africa shares three-quarters of the global malaria cases with sub-Saharan Africa carrying the largest burden (35.4 million disability adjusted life years) [2].

Located in the horn of Africa, Eritrea is topographically classified as western and eastern lowlands and central highlands. Most regions of the country receive low sporadic rainfall, with a significant difference in rainfall amount between the highland and lowland areas. The coastal plains (0–1000 meters above sea level) have very similar malaria situation as the western lowlands, but with notably less precipitation. In the western lowlands (with a range of elevation between 700 and 1500 meters above sea level), malaria transmission is highly seasonal and the area is prone to epidemics. Transmission is perennial along the rivers, valleys, dams, and irrigation projects. The highlands (>1500 meters above sea level) are generally free from malaria but are highly prone to malaria epidemics as a result of low immunity of these populations. With an estimated population size of four million, about 46.5% of the Eritrean population lives in the zones classified as moderate malaria-risk areas. The most common malaria parasite in Eritrea is *Plasmodium falciparum* which accounts for more than 80% of all malaria cases. *Plasmodium vivax* is the second species which accounts for about 20%. *Anopheles arabiensis* is the main vector of malaria in the country followed by *A. dthali*, *A. cinereus*, *A. rhodesiensis*, *A. squamosus*, and *A. rupicolus* [3]. In Eritrea, there are six administrative regions (zones) with 58 districts (subzones). In each zone, the socioeconomic drivers of malaria transmission are well known; in fact, each zonal and/or subzonal malaria coordinator knows the vector breeding sites in the locality. The drivers of malaria transmission in each of the zones are varied. In the Gash Barka zone, the area has a July-September rainfall season and a small rain season in April/May. The drivers of transmission are the many brick-making sites, the big rivers, the borders with Ethiopia and Sudan, many inaccessible areas, lots of dams, and irrigation activities. This is coupled with movement of nonimmune people to these areas for economic activities which may worsen the malaria situation. In the Debub zone, months July-September are the rainy seasons and the April/May small rain. Drivers of transmission are similar to those in Gash Barka. It also shares border with Ethiopia, but limited movement of people across the boarders limits the cross-border transmission of malaria here. Debub has the highest population density in the country. Anseba zone has July-September rainy season and the April/May small rain. This zone has imported malaria cases from Gash Barka. There are lots of dams and rivers. The zone has good surveillance system, and it reports that 60% of malaria cases in the zone are imported from Gash Barka. Semenawi Keih Bahri zone has December/January raining season with no April/May small rain. The land mass is very big, but malarious areas are limited because the coastal area is desert and very hot. It has rivers and two raining seasons in some areas. Debubawi Keih Bahri zone has December/January rainy season and no April/May small rain; there is perhaps no local transmission, and cases are imported from the western lowland and southern region. Despite the reduction in malaria morbidity and mortality, the disease remains a public health concern in most parts of the country threatening economic development. The disease is most prevalent in Gash Barka and Debub zones, which bear more than 85% of the national burden.

Over the past two decades, successful implementation of various strategies to combat malaria has led to a significant decrease in malaria incidence in Eritrea [4–8]. In 2012, there was 89% reduction in malaria incidence from 157 cases/1000 population at risk in 1998 to 17 cases/1000 in 2016. In the same year, there was a 98% reduction (0.004 deaths/1000) in malaria-specific deaths compared to 0.198 deaths/1000 in 1998. In some parts of Eritrea, there seems to be a “break in malaria transmission” as subzones that previously reported thousands of cases are reporting very few or nil cases. Furthermore, the NMCP has developed a National Malaria Strategic Plan to eliminate malaria in Eritrea by 2030 [9, 10].

Malaria stratification is classification of areas according to the risk of malaria. Malaria risk mapping is a useful preliminary stage to differentiate areas that experience epidemic or highly seasonal transmission of malaria. In moderate and low transmission settings characterized by marked spatial heterogeneity of malaria risk, the stratification level needs to be more spatially specific in order to facilitate precise targeting of interventions, ideally at the subdistrict or village level [11–13]. Stratification, spatiotemporal distribution, and disease surveillance for the subzone level early detection of malaria epidemics has been used in many countries [14–18]. In Eritrea, malaria control programs are designed to suit at the level of administrative zones (subzones). Thus, the subzone level stratification is more appropriate than large-scale stratification, which may cause overlapping of strata.

Eritrea is an example of a country that has achieved substantial reductions in malaria morbidity and mortality and is now making a shift from a “control” phase to the “preelimination” phase. The National Strategic Plan has set a goal to reduce malaria incidence by 50% from 2010 and achieve the test positivity rate (TPR) below 5% in all subzones by 2017 and beyond. To attain this goal, one part of the objectives aims to strengthen the malaria surveillance system in light of preelimination and detect and respond to 100% of malaria epidemics within two weeks of onset [19]. Consequently, the NMCP has been making efforts in targeting malaria interventions through a more refined malaria stratification risk approach [20, 21] and to provide timely data on temporal and spatial variations in malaria case numbers across all malaria-endemic subzones in the country [22].

The effects of climate change on malaria remain complex [23–25]. Various studies have argued that climatic conditions alone are not responsible for the observed changes in malaria transmission, but other socioeconomic factors such as land use change, population growth, migration changes, and economic development play a considerable role in the disease transmission dynamics [26]. Nevertheless, many studies found a link between changes in climatic factors and malaria transmission [27–36]. Some researchers posit that both explanations are plausible since various factors complement and interact with each other at different time scales [27]. Recent studies have found relationships of climate variables, typically temperature and rainfall, with malaria incidence. For instance, links between temperature and malaria transmission have been observed in many countries [31, 37–39]. Relationships between rainfall and malaria transmission have also been reported in many studies worldwide [31, 37–40].

Because rainfall created wet conditions suitable for breeding, vector coverage and disease season unpredictability is common. It is worth nothing that an increase in rainfall does not necessarily increase in malaria cases and vice versa. Heavy rainfall tends to be associated with the detrimental effect in breeding sites, while moderate rainfall may favor vector abundance [27, 40].

An understanding of the trends and relationships between malaria transmission and rainfall could be beneficial for anticipating disease epidemics and making early preparations for prevention and control. While the relationships between the malaria distribution and environmental condition have been studied to some extent, the effects of rainfall on malaria incidence have not been studied in Eritrea. Lack of information on the expected number of malaria cases in a given area in a certain period of time is a challenge to the NMCP because of the difficulties to predict “when and where epidemics will occur”. Hence, the development of a simple prediction model to forecast the occurrence of malaria transmission by rainfall variability offers an opportunity for early detection of malaria epidemics for a timely response. However, there have been no studies that provide forecasted malaria incidence based on rainfall in Eritrea. In this context, this study aimed at constructing a national malaria stratification map and developing models and forecast malaria incidence in Eritrea based on rainfall variability.

## 2. Methodology

### 2.1. Study Design

This is a retrospective analytic cross-sectional study.

### 2.2. Study Area

Eritrea is a malaria-epidemic-prone country situated in the Horn of Africa with an approximate area of 124,000 square kilometers (sq km). The study was conducted in all six zones with 58 subzones of the country.

### 2.3. Study Population

Retrospective census analysis of all health facility reports of malaria cases from 2012 to 2016 from the National Health Management and Information System (NHMIS) office.

### 2.4. Data Collection Method

#### 2.4.1. Data on Malaria Cases

All malaria cases reported from health facilities to NHMIS were extracted by month and health facility for the years 2012–2016. Zonal and subzonal boundary files were obtained from the National Statistics Office (NSO), Asmara, Eritrea. Malaria cases were summed over both age groups (under and above 5 years) by the subzone and month for the given number of years. Malaria incidence per 100,000 persons per month by the subzone was calculated by using the year 2015 population estimates obtained from the National Statistics Office.

#### 2.4.2. Rainfall Data

From 2012–2016, monthly rainfall data from all ground metrological stations in Eritrea were collected from the National Civil Aviation, Ministry of Agriculture, Ministry of Water, Land and Environment and Eritrea National Mapping and Information Center. Comparison of data quality and completeness was checked, and the most reliable data were selected from the Ministry of Agriculture.

### 2.5. Data Analysis

#### 2.5.1. Spatial Distribution Analysis (Stratification Map)

Monthly malaria incidence during the 5-year period for all subzones was used for stratification. Stratification map was constructed using nonhierarchical cluster analysis (using the *K*-means cluster analysis menu in SPSS). Validity of the stratified clusters was checked by examining the maximum absolute coordinate change and one-way analysis of variance (ANOVA) with a conservative post hoc test using Bonferroni as the multiple comparisons test to determine the difference of mean incidence rates between the constructed clusters. Finally, each cluster was marked with a different color on the subzone level map.

#### 2.5.2. Autocorrelation and Cross-Correlation Analysis

Using the Ljung–Box Q test, the autocorrelation coefficient (AC), and partial autocorrelation coefficient (PAC), the effect of lagged months on malaria incidence was assessed. A cross-correlation analysis was also conducted to detect how malaria transmission was influenced by up to seven lagged months.

#### 2.5.3. Time-Series Models

The forecasting menu of IBM SPSS Statistics 20 was used for data analysis. The dependent variable was the monthly malaria cases of the stratified clusters using the 5 years malaria incidence data (2012–2016). The independent variable was the total rainfall (in mm). Expert modeler was used to build the model. Appropriate date variables were defined for the consideration of seasonal terms in the model by using the define date menu from the transform tab in SPSS.

In order to take into account the different situations that may bias classical regression models, the monthly malaria incidence and rainfall modeling was performed using a seasonal autoregressive integrated moving average (SARIMA) model. The simplified notation for seasonal ARIMA is ARIMA (*p*, *d*, *q*) × (*P*, *D*, *Q*)_12_, where *p* indicates the nonseasonal autoregressive (AR) order, *d* is the nonseasonal differencing, and *q* indicates the nonseasonal moving average (MA) order. *P*, *D*, and *Q* are the corresponding seasonal components. *S* indicates the period, which in this case is 12 months.

Normalized Bayesian Information Criteria (N.BIC) and stationary *R*-squared (coefficient of determination) indicators were used to determine the fitness of the model, and the Ljung-Box Q (LB) statistic was used to determine its suitability. Models with a LB significance value of more than 0.05 were considered suitable. Finally, models with the best fit, with respect to the above criteria, were selected. The files of the selected models were stored as XML data and applied for forecasting the 2017 malaria incidence rates. Model validity was checked by examining the relationship of the observed and predicted incidence using the Spearman correlation coefficient and testing the degree of difference of the observed and predicted monthly malaria incidences using the paired samples *t*-test.

## 3. Results

### 3.1. Spatial Distribution of Malaria in Eritrea

There were 87,666 reported malaria cases in Eritrea from 2012 to 2016. No malaria cases were reported in Areta and central Denkalia subzones during this period. The highest malaria incidences were mainly distributed in Gash Barka and Debub subzones, namely, May Ayni (5086/100, 000 population), May Mine (6271), Goluj (6417), Molqui (6744/100, 000), Barentu (7138/100, 000), Teseney (7861/100, 000), Ghindae (11302/100, 000), and Mogolo (14324/100, 000).

### 3.2. Subzone Stratification into Clusters by Nonhierarchical Clustering Analysis

**Figure 1:**
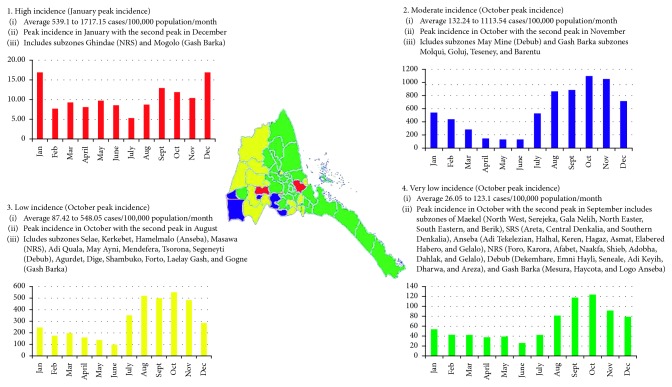
Eritrea malaria stratification map by monthly malaria incidence at subzone levels.

A four cluster map was found to be the most valid of the reported malaria incidence during the study period. With these clusters, we were able to capture differences in both intensity and the seasonal dynamics of malaria incidence. As shown in Figure 1, the subzones were stratified according to the level of incidence rates by nonhierarchical clustering.  Table 1 shows the detailed list of subzones categorized with their respective clusters.

### 3.3. Validity Test of the Stratified Subzone Clusters

Ideally, the recommended number of clusters for stratification is 3 to 5. However, in this study, three cluster stratification was not suitable because in the iteration history (change in cluster centers), only one cluster showed the maximum absolute coordinate change. The five cluster stratification was not applied because, although all clusters showed the maximum absolute coordinate change, there was no significant difference between clusters four and five in the post hoc test. Hence, the four cluster stratification was used. Validity of this clustering was checked by looking at the maximum absolute coordinate change, and all clusters had a coordinate center of 0.001. Validity was also performed by one-way ANOVA with a post hoc test using a conservative Bonferroni test to determine the difference of mean incidence rates between the four clusters. As shown in Table 2, there was strong significant difference between all clusters. (*P* < 0.001).

### 3.4. Autocorrelation and Partial Autocorrelation of Monthly Malaria Incidence of Stratified Clusters

In all clusters, the *P* value of the Ljung-Box Q Statistic of each lagged month was less than 0.05. In the high-risk cluster, the absolute values of the autocorrelation and partial autocorrelation coefficients showed strong associations during the first three lagged months and the first two lagged months of rainfall for the PAC. In the moderate-risk cluster, there was a strong autocorrelation of monthly malaria incidence during the first two lagged months for the AC and first five months for the PAC was found. For the low-risk cluster, there was a strong autocorrelation of monthly malaria incidence during the first three lagged months for the AC and first two months for the PAC. Similarly, in the very low-risk cluster, there was a strong autocorrelation of monthly malaria incidence during the first three lagged months for the AC and first two months for the PAC.

### 3.5. Cross-Correlation between Monthly Malaria Incidence and Rainfall

Cross-correlation analyses showed that the monthly incidence of malaria in the high-risk cluster was not associated with monthly precipitation. In moderate-risk cluster, the monthly incidence of malaria was correlated with previous 1, 2, 3, and 4 lagged months of rainfall. In low-risk cluster, the monthly incidence of malaria was correlated with previous 2, 3, and 4 lagged months of rainfall, whereas in the very low cluster, with previous 1, 2, and 3 lagged months (Table 3).

### 3.6. Time-Series Analysis

#### 3.6.1. Model Building

Using data from 2012 to 2016, the monthly malaria incidence and rainfall modeling was performed using a seasonal autoregressive integrated moving average (SARIMA) model.

With model type of ARIMA (1, 0, 0) (0, 0, 0), the model for the high-risk cluster fitted well as it explained 73% of the variability of the monthly malaria incidence by rainfall. The Normalized Bayesian Information Criteria also showed the model was a good fit for the given data. The Ljung-Box test for this model was not significant (LB = 14.218, *p*=0.652), indicating suitability of the model. In the moderate-risk cluster, a simple seasonal adjustment model fairly fitted as the model explained 43.3% of the variability of the monthly malaria incidence by rainfall. The Normalized Bayesian Information Criteria (8.462) showed the model was a good fit for the given data. The model suitably fitted the data as the Ljung-Box test for this model was not significant (LB = 21.899, *p*=0.146). Similarly, the models for the low-risk and very low-risk clusters fairly fitted the data with the nonsignificant Ljung-Box value.  Table 4 shows details of the fitted (SARIMA) model of malaria prevalence and rainfall of the stratified clusters.

#### 3.6.2. Model Validity

Model validity was done by building a model using the 2012–2016 data and tested the predicted incidence of 2016 with its actual (observed) values using Pearson's correlation coefficient and paired samples *t*-test. The Pearson's correlation coefficient showed statistically significant correlation between the predicted and observed incidences. The *t*-test for all clusters was nonsignificant, indicating the malaria incidence of each month in the observed and predicted incidences was not statistically significant (Table 5).

**Figure 2:**
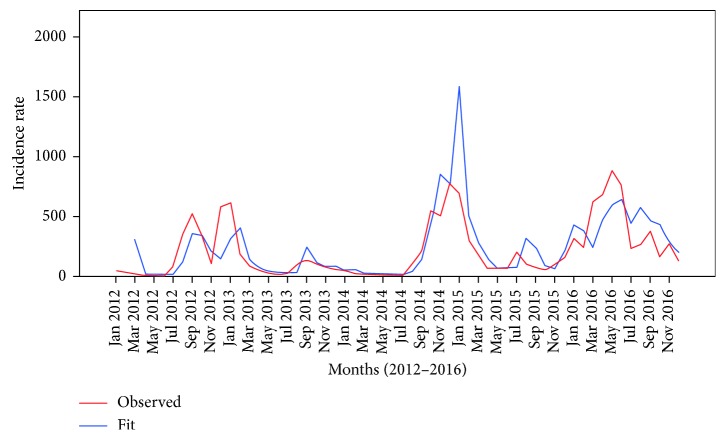
Graphs of the observed and predicted cases for high-risk cluster prediction model.

Figure 2 shows predicted cases comparing with the observed cases in the best-fit model of the high-risk cluster. In this model, the predictions made in the first year were less than the actual number of observed cases. The number of cases predicted at the end of the third year (2014) and first-quarter of the next year was higher than the actual number of cases.

**Figure 3:**
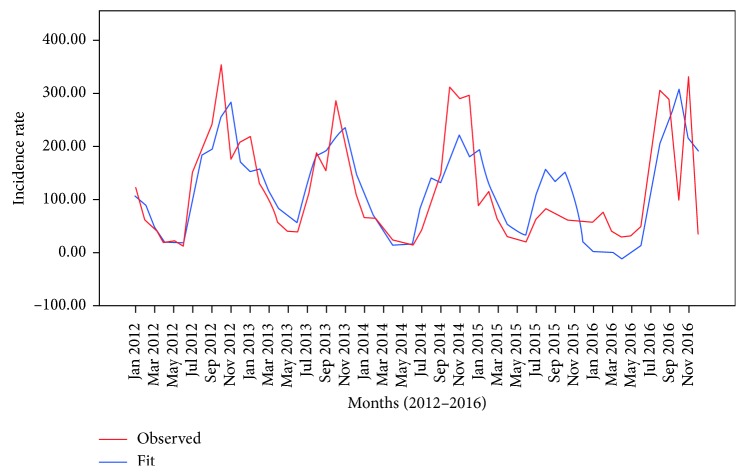
Graphs of the observed and predicted cases for the moderate-risk cluster prediction model.

Figure 3 shows predicted cases comparing with the observed cases in the best-fit model of the moderate-risk cluster. In this model, the predictions made in this model were slightly lower than the observed cases except in the fourth-quarters of the fourth year.

**Figure 4:**
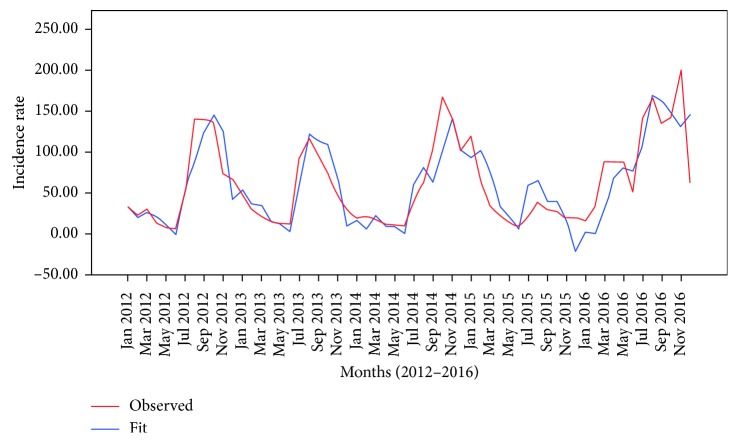
Graphs of the observed and predicted cases for the low-risk cluster prediction model.

Figure 4 shows predicted cases comparing with the observed cases in the best-fit model of the low-risk cluster. In this model, the predictions made were roughly similar with the observed cases except in the third-quarters of the fourth year and first-quarter of the fifth year.

**Figure 5:**
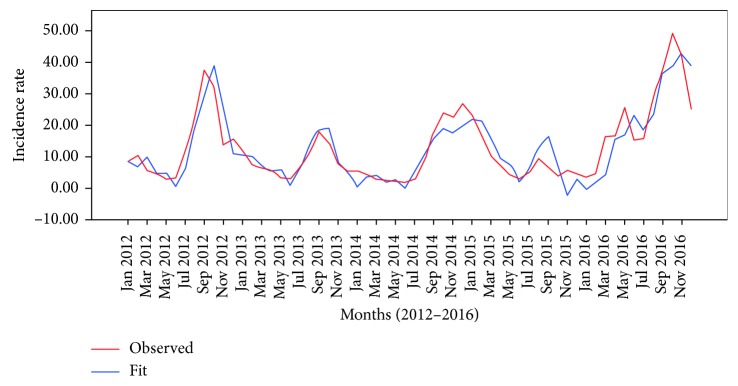
Graphs of the observed and predicted cases for the very low-risk cluster prediction model.

Figure 5 shows predicted cases comparing with the observed cases in the best-fit model of the very low-risk cluster. Predictions made were roughly similar with the observed cases except in the third-quarters of the fourth year and first-quarter of the fifth year.

#### 3.6.3. Forecasting Malaria Incidence of Clusters for the Years 2017 and 2018

The SARIMA models constructed were used to make future forecasting of malaria incidence rates for the year 2017 (Table 6).

## 4. Discussion

Using a five-year surveillance data, this study described the malaria risk stratification at a subzone level and explored the potential effect of rainfall on malaria incidence in Eritrea.

Nationally, the malaria incidence showed a decreasing trend during 2012–2016. The results of our study confirmed that the malaria intensity and seasonality in Eritrea vary in different areas and during different years, which was consistent with other studies [27, 41, 42]. Early attempts to stratify malaria incidence for Eritrea have been made by Ceccato et al. using the 1998–2003 malaria case reports. In their study, they reported a five cluster map as an ideal stratification system and identified distinct seasonality and malaria dynamics at subzone levels—where most of them were in the high malaria-risk area category [20]. Our study made an update of this map and found out that the number of malaria incidence has reduced significantly. Consequently, the number of subzones who were categorized in the high- and moderate-risk areas was now under the “very low-risk category”. Investing in the high burden, poorly served, or densely populated areas aids to direct limited resources to where they are needed for a more efficient malaria intervention to reach different measurable milestones. In this regard, Omumbo et al. noted that unless stratification information is used to ensure the evidence-based added value in planning control strategies, and for a more rational basis of financing, the successes of the recent investment in malaria control may be lost [43]. Hence, our findings indicate that public health resource allocation should focus on the areas and months with the highest malaria risk.

In this study, rainfall was found to be an important meteorological factor for prediction of malaria incidence. The SARIMA model was used based on biological consideration because rainfall can affect malaria transmission not only in the same month as the rainfall occurs but also in several continuous months. Except in the high-risk cluster subzones, all of the constructed models followed a simple seasonal adjustment method. After testing five methods using incidence data in Ethiopia, Abeku T. and his colleagues argue that a seasonal adjustment method produces the best forecasts and concludes that forecasting incidence from historical morbidity patterns alone have limitations and underscores the need for incorporating external predictors such as meteorological factors in model building [44].

The modeling results in this study showed that 73.1%, 46.3%, 53.4%, and 50.7% of the variance in malaria transmission were explained by rainfall in the high-risk, moderate-risk, low-risk and very low-risk clusters, respectively. Because of the high cross-correlations found between rainfall and malaria time-series data, this study adds to the argument that rainfall is a driver of malaria seasonality. Similarly, a study conducted by Devi and Jauhari [45] suggested that monthly malaria incidence is related to rainfall changes. Using a simple linear regression, one study from Burundi found negative correlation of malaria incidence and rainfall on similar months [46]. However, since the researchers assumed a simple linear correlation between malaria epidemics and climate factors without testing the linearity of the relationship, these conclusions have been questioned by some researchers [47]. In many studies, positive correlations between rainfall and malaria incidence have been reported [47–51]. However, due to the complex relationship between the rainfall and entomological variables and malaria incidence, the efforts to build statistical models using rainfall to predict malaria have not been successful in many cases [18, 52–54].

Considerable variation on incidence of malaria and rainfall variability was observed between time interval (lag time) of the present study and similar other studies. In most studies, the impact of this variable has been correlated with a monthly lag time [55, 56], one to two months lag time [40, 42, 57], and one to 9 weeks lag time [42]. Although a causal relationship is biologically plausible at a lag of two to four months, it is increasingly less at longer lag times [58]. Yet, some studies found significant relationship even at six to seven lagged months [59]. However, in this study, the highest positive correlation between rainfall and malaria was seen with a lag time of 2, 3, and 4 months. These variations could be associated with the difference between the climatic conditions, differences in malaria incidence rates, climate variables examined, and number of years used for modeling. Also, these studies are difficult for comparison as they have been conducted in varied geographic, climatic conditions, source of climatic data, type of parasite under investigation, and endemicity contexts. Sources of climate data and area coverage were also varied in different studies. In this study, rainfall data were obtained from ground meteorological stations. The past temporal records of the number of malaria cases are the most important variables of the models in this study. These variables were significant in the monthly cluster models and have had a considerable effect on model fit. In fact, the number of previous malaria cases may reflect the interaction of all the current factors effective in malaria incidence, including meteorological, social, and economic variables.

Due to some limitations, the findings of the present study should be interpreted with caution. First, the time period used to build the model in this study was relatively short and apart from rainfall data, and other important meteorological variables like temperature and relative humidity were not available for model building. Despite these limitations, this study can be considered as a step towards clarifying the relationship between rainfall and incidence of malaria in Eritrea and statistical models to consider for future prediction.

## 5. Conclusion

The spatial distribution of malaria varied in different subzones during the study period. Rainfall was an important meteorological factor for prediction of malaria incidence. The highest positive correlation between rainfall and malaria was seen with 2, 3, and 4 lagged months of rainfall. The modeling results showed that 73.1%, 46.3%, 53.4%, and 50.7% of the variance in malaria transmission was explained by rainfall in the high-risk, moderate-risk, low-risk and very low-risk clusters, respectively. Thus, the statistical models can be used in line with a Malaria Early Warning System to predict malaria incidence. Further research considering modeling of malaria incidence on other meteorological variables like temperature and relative humidity is recommended. Further modeling and forecasting of malaria incidence should be implemented at a village or health facility level.

## Figures and Tables

**Table 1:** Stratification of subzones by nonhierarchical clustering.

Cluster number	Subzones	Malaria incidence M^*∗*^ (IQR)^*∗∗*^
High incidence rate	Ghindae and Mogolo	99.65 (265.675)
Moderate incidence rate	May Mine, Molqui, Goluj, Teseney, and Barentu	73.79 (139.515)
Low incidence rate	Selae, Kerkebet, Hamelmalo, Masawa, Adi Quala, May Ayni, Mendefera, Tsorona, Segeneyti, Agurdet, Dige, Shambuko, Forto, Laelay Gash, and Gogne	40.71 (75.755)
Very low incidence rate	North West, Serejeka, Gala Nefih, North Easter, South Eastern, Berik, Areta, Central Denkalia, Southern Denkalia, Adi Tekelezan, Halhal, Keren, Hagaz, Asmat, Elabered, Habero, Geleb, Foro, Karora, Afabet, Naakfa, Shieb, Adobha, Dahlak, Gelalo, Dekemhare, Emni Hayli, Seneafe, Adi Keyih, Dbarwa, Areza, Mesura, Haycota, and Logo Anseba	8.49 (12.997)

^*∗*^Median; ^*∗∗*^interquartile range.

**Table 2:** Comparison of mean difference of incidence rates by clusters using the post hoc test (Bonferroni as multiple comparisons).

Cluster	Comparison cluster	Mean difference	Significance	95% confidence interval	
				Lower bound	Upper bound
High risk	Moderate risk	3215.37^*∗*^	<0.001	2165.4269	4265.3065
	Low risk	6117.46^*∗*^	<0.001	5143.6194	7091.3006
	Very low risk	−5927.09^*∗*^	<0.001	−7628.1869	−4225.9931

Moderate risk	Low risk	2902.09^*∗*^	<0.001	2271.8722	3532.3145
	Very low risk	−9142.46^*∗*^	<0.001	−10672.9938	−7611.9195

Low risk	Very low risk	−12044.55^*∗*^	<0.001	−13523.9201	−10565.1799

^*∗*^Mean difference is significant at *p* < 0.001.

**Table 3:** Cross-correlation analysis by cluster with CCF and SE.

	High-risk cluster		Moderate-risk cluster		Low-risk cluster		Very low-risk cluster	
Lagged months	CCF	SE	CCF	SE	CCF	SE	CCF	SE
−7	0.017	0.137	−0.159	0.137	−0.218	0.137	−0.140	0.137
−6	0.188	0.136	−0.020	0.136	−0.093	0.136	−0.016	0.136
−5	0.318^*∗*^	0.135	0.147	0.135	0.046	0.135	0.182	0.135
−4	0.305^*∗*^	0.134	0.365^*∗*^	0.134	0.308^*∗*^	0.134	0.389^*∗*^	0.134
−3	0.297^*∗*^	0.132	0.585^*∗*^	0.132	0.566^*∗*^	0.132	0.556^*∗*^	0.132
−2	0.300^*∗*^	0.131	0.474^*∗*^	0.131	0.575^*∗*^	0.131	0.567^*∗*^	0.131
−1	0.282	0.130	0.290^*∗*^	0.130	0.451^*∗*^	0.130	0.365^*∗*^	0.130
0	0.029	0.129	0.066	0.129	0.211	0.129	0.009	0.129
1	−0.007	0.130	−0.284^*∗*^	0.130	−0.141	0.130	−0.266^*∗*^	0.130
2	−0.043	0.131	−0.492^*∗*^	0.131	−0.358^*∗*^	0.131	−0.323^*∗*^	0.131
3	−0.153	0.132	−0.506^*∗*^	0.132	−0.341^*∗*^	0.132	−0.262^*∗*^	0.132
4	−0.061	0.134	−0.392^*∗*^	0.134	−0.271^*∗*^	0.134	−0.190	0.134
5	−0.099	0.135	−0.182	0.135	−0.211	0.135	−0.142	0.135
6	−0.108	0.136	−0.028	0.136	−0.139	0.136	−0.080	0.136
7	0.028	0.137	0.146	0.137	−0.041	0.137	0.020	0.137

CCF: cross-correlation coefficient; SE: standard error; lag: the number of lagged months. ^*∗*^Significant cross-correlation of the lagged month between the monthly malaria incidence and rainfall.

**Table 4:** Best-fitted seasonal autoregressive integrated moving average (SARIMA) model of malaria incidence and rainfall of the stratified clusters.

Cluster	SARIMA model	Model fit		Model diagnosis (Ljung-Box Q test)	
		S.R^2^	N.BIC	Value	Significance
High risk	ARIMA (1, 0, 0) (0, 0, 0)	0.731	10.806	14.218	0.652
Moderate risk	Simple seasonal	0.463	8.462	21.899	0.146
Low risk	Simple seasonal	0.534	6.787	21.029	0.177
Very low risk	Simple seasonal	0.507	3.377	15.833	0.465

S.R^2^: stationary *R*-squared (coefficient of determination); N.BIC: Normalized Bayesian Information Criteria.

**Table 5:** Cluster validity test of the best-fitted model of malaria incidence and rainfall.

Cluster	SARIMA model	Model validity				
		Pearson's correlation		Paired samples *t*-test		
		Value	Significance	Value	df	Significance
High risk	ARIMA (1, 0, 0) (0, 0, 0)	0.597^*∗*^	0.041	0.036	11	0.972
Moderate risk	Simple seasonal	0.647^*∗*^	0.023	−0.688	11	0.506
Low risk	Simple seasonal	0.746^*∗*^	0.005	−0.586	11	0.570
Very low risk	Simple seasonal	0.873^*∗*^	0.001	−0.809	11	0.436

^*∗*^Significant at *P* < 0.05.

**Table 6:** Cluster level malaria incidence forecast for the year 2017.

Forecasted malaria incidence per 100,000	High-risk cluster subzones	Moderate-risk cluster subzones	Low-risk cluster subzones	Very low-risk cluster subzones
January	131.30	74.11	71.36	20.28
February	0	43.17	58.55	18.50
March	0	16.1	62.30	18.02
April	0	12.8	54.11	17.12
May	0	12.17	50.76	17.38
June	0	91.88	41.10	15.01
July	0	159.41	93.08	18.20
August	0	165.69	128.85	25.93
September	51.97	208.43	124.73	33.23
October	32.19	198.04	133.22	34.42
November	0	128.59	120.24	28.07
December	128.02	74.11	79.31	25.25

## Data Availability

All data supporting the conclusions of this study are available upon reasonable request.

## Abbreviations

ACF: Autocorrelation coefficient
ANOVA: Analysis of variance
CCF: Cross-correlation coefficient
HMIS: Health management information system
NBIC: Normalized Bayesian Information Criteria
NDVI: Normalized difference vegetation index
NMCP: National Malaria Control Program
PACF: Partial autocorrelation coefficient
RH: Relative humidity
SARIMA: Seasonal autoregressive moving average
TPR: Test positivity rate
WHO: World Health Organization.
